# Hydrogen sulfide-releasing cyclooxygenase inhibitor ATB-346 enhances motor function and reduces cortical lesion volume following traumatic brain injury in mice

**DOI:** 10.1186/s12974-014-0196-1

**Published:** 2014-12-04

**Authors:** Michela Campolo, Emanuela Esposito, Akbar Ahmad, Rosanna Di Paola, Irene Paterniti, Marika Cordaro, Giuseppe Bruschetta, John L Wallace, Salvatore Cuzzocrea

**Affiliations:** Department of Biological and Environmental Sciences, University of Messina, Viale Ferdinando Stagno D’Alcontres, 31-98166 Messina, Italy; Inflammation Research Network, University of Calgary, 3330 Hospital Drive NW, Calgary, Alberta T2N 4 N1 Canada; Manchester Biomedical Research Centre, Manchester Royal Infirmary, School of Medicine, University of Manchester, 29 Grafton Street Manchester, M13 9WU Manchester, UK

**Keywords:** Brain trauma, Hydrogen sulfide, Neurotrophic factor, Inflammation, Motor recovery, Infarct area, Infarct volume, Nitrosative stress, Astrogliosis, Neuroprotection

## Abstract

**Background:**

Traumatic brain injury (TBI) induces secondary injury mechanisms, including dynamic interplay between ischemic, inflammatory and cytotoxic processes. We recently reported that administration of ATB-346 (2-(6-methoxynapthalen- 2-yl)-propionic acid 4-thiocarbamoyl-phenyl ester), a hydrogen sulfide-releasing cyclooxygenase inhibitor, showed marked beneficial effects in an animal model of spinal cord injury, significantly enhancing recovery of motor function and reducing the secondary inflammation and tissue injury.

**Methods:**

Here we evaluated the neuroprotective potential of ATB-346, a hydrogen sulfide-releasing derivative of naproxen, using the controlled cortical impact (CCI) injury model in mice, one of the most common models of TBI. Moreover, the aim of the present study was to carefully investigate molecular pathways and subtypes of glial cells involved in the protective effect of ATB-346 on inflammatory reaction associated with an experimental model of TBI. In these studies, TBI was induced in mice by CCI and mice were orally administered ATB-346, naproxen (both at 30 μmol/kg) or vehicle (dimethylsulfoxide:1% carboxymethylcellulose [5:95] suspension) one and six hours after brain trauma and once daily for 10 days.

**Results:**

Results revealed that ATB-346 attenuated TBI-induced brain edema, suppressed TBI-induced neural cell death and improved neurological function. ATB-346 also significantly reduced the severity of inflammation and restored neurotrophic factors that characterized the secondary events of TBI.

**Conclusions:**

These data demonstrate that ATB-346 can be efficacious in a TBI animal model by reducing the secondary inflammation and tissue injury. Therefore, ATB-346 could represent an interesting approach for the management of secondary damage following CNS diseases, counteracting behavioral changes and inflammatory process.

## Background

Traumatic brain injury (TBI) is a growing public health concern worldwide. There are over 1.35 million emergency room visits and 275,000 hospitalizations for nonfatal TBI each year in the United States, and approximately 40% of these individuals suffer from long-term disability due to their injury [1]. The pathophysiology of TBI can be divided into primary and secondary brain injury [2]. Primary injury results from the direct, physical brain trauma with tissue distortion, shearing, vascular injury and cell destruction probably related to rotational acceleration and deceleration inertial forces. Secondary brain injury is related to destructive inflammation and biochemical changes. Secondary injury onsets within minutes of primary injury, may last for several days and contributes to final outcome [3]. Primary and secondary brain injuries induce cerebral edema and bleeding. During secondary neuronal injury, healthy neurons around the injury site progressively degenerate, eventually leading to more serious clinical symptoms. Therefore, secondary neuronal injury plays a key role in the severity of insult and subsequent clinical prognosis.

Non-steroidal anti-inflammatory drugs (NSAIDs) are among the most commonly used anti-inflammatory drugs, but their use is associated with significant, sometimes life-threatening, adverse effects, particularly in the gastrointestinal (GI) tract [4]. Along with nitric oxide (•NO) and carbon monoxide (CO), hydrogen sulfide (H_2_S) is regarded as an important gasotransmitter and endogenous neuromodulator, drawing increasing attention in the literature. Traditional neurotransmitters bind and activate membrane receptors, while gasotransmitters can freely diffuse to adjacent cells and directly bind to their target proteins to modify biological functions. Therefore, H_2_S is a physiologic gasotransmitter as important as •NO and CO. In the past decade, increasing evidence shows that H_2_S plays multiple roles in the CNS under physiological and pathological states. H_2_S is produced in various parts of the body including the heart [5], the cardiovascular system [6] and the central nervous system (CNS) [7]. With respect to the CNS, H_2_S has been reported to exert neuroprotective and neuromodulatory effects [8,9]. Thus, H_2_S has recently been exploited in the design of novel NSAID derivatives that exhibit little, if any, side effects in the GI tract, despite producing suppression of prostaglandin synthesis and reduction of inflammation at least as effectively as the parent NSAID [10]. Recently, beneficial effects of an H_2_S-releasing derivative of naproxen have been shown in an animal model of spinal cord injury (SCI), significantly enhancing recovery of motor function, possibly by reducing the secondary inflammation and tissue injury that characterizes this model. The combination of inhibition of cyclooxygenase [11] and delivery of H_2_S may offer a promising alternative to existing therapies for traumatic injury [12]. On the basis of these data, H_2_S could have an important role in reducing inflammatory processes and tissue damage post-brain trauma. Therefore, in the current study we evaluated ATB-346, a novel H_2_S-releasing derivative of naproxen, for neuroprotective properties in experimental murine TBI using controlled cortical impact injury (CCI), a model of focal brain injury. Moreover, the aim of the present study was to carefully investigate molecular pathways and subtypes of glial cells involved in the protective effect of ATB-346 on inflammatory reaction associated with an experimental model of TBI. In particular, our attention shifts to post-injury recovery of motor function, reduction of infarct area and of brain tissue inflammation after TBI.

## Methods

### Animals

Male CD1 mice (25 to 30 g, Harlan, Milan, Italy), aged between 10 and 12 weeks, were used for all studies. Mice were housed in individual cages (five per cage) and maintained under a 12:12 hour light/dark cycle at 21 ± 1°C and 50 ± 5% humidity. Standard laboratory diet and tap water were available *ad libitum*. The study was approved by the University of Messina Review Board for the care of animals. Animal care was in compliance with Italian regulations on protection of animals used for experimental and other scientific purposes (Ministerial Decree 16192) as well as with the Council Regulation (EEC) (Official Journal of the European Union L 358/1 12/18/1986).

### Controlled cortical impact experimental traumatic brain injury

TBI was induced in mice by a controlled cortical impactor. The mice were anesthetized under intraperitoneal ketamine and xylazine (2.6/0.16 mg/kg of body weight, respectively). A craniotomy was made in the right hemisphere, encompassing bregma and lambda, and between the sagittal suture and the coronal ridge, with a Micro motor hand piece and drill (UGO Basile SRL, Comerio Varese, Italy). The resulting bone flap was removed and the craniotomy enlarged further with cranial rongeurs (New Adalat Garh, Roras Road, Pakistan**)**. A cortical contusion was produced on the exposed cortex using the controlled impactor device Impact One^TM^ Stereotaxic impactor for CCI (Leica, Milan, Italy). Briefly, the impacting shaft was extended, and the impact tip was centered and lowered over the craniotomy site until it touched the dura mater. Then, the rod was retracted and the impact tip was advanced farther to produce a brain injury of moderate severity for mice (tip diameter: 4 mm; cortical contusion depth: 3 mm; impact velocity: 1.5 m/sec). Immediately after injury, the skin incision was closed with nylon sutures, and 2% lidocaine jelly was applied to the lesion site to minimize any possible discomfort.

### Test drugs

ATB-346 (2-(6-methoxynapthalen-2-yl)-propionic acid 4-thiocarbamoyl phenyl ester) is a derivative of naproxen, which includes a H_2_S-releasing moiety referred to hereafter as ‘TBZ’ (4-hydroxythiobenzamide) [13]. ATB-346, TBZ and naproxen were prepared freshly each day as suspensions in dimethylsulfoxide:1% carboxymethylcellulose (5:95).

Prior to beginning these experiments, a pilot study was performed to confirm the equipotency of naproxen and ATB-346 in suppressing cyclooxygenase at the dose selected. At 30 μmol/kg (oral administration), naproxen and ATB-346 equally suppressed gastric (prostaglandin E2) PGE_2_ synthesis (by more than 90%) and whole blood thromboxane synthesis ( by more than 95%). This level of inhibition was evident within 15 minutes and persisted for at least 12 hours after drug administration. TBZ had no effect on gastric PGE_2_ synthesis or whole blood thromboxane synthesis.

### Experimental groups

Mice were randomly allocated into one of five groups. In the TBI + vehicle group, mice were subjected to TBI and received the vehicle for TBZ, naproxen and ATB-34 (dimethylsulfoxide:1% carboxymethylcellulose) (orally), at one and six hours after brain trauma (N = 20). The TBZ group was the **s**ame as the TBI + vehicle group, but mice were administered TBZ only (30 μmol/kg, orally), at one and six hours after brain trauma (N = 20). The naproxen group was the same as the TBI + vehicle group, but mice were administered naproxen only (30 μmol/kg, orally), at one and six hours after brain trauma (N = 20). The ATB-346 group was the same as the TBI + vehicle group, but mice were administered ATB-346 only (30 μmol/kg, orally), at one and six hours after brain trauma (N = 20). In the sham + vehicle group mice were subjected to identical surgical procedures, except for TBI, and were kept under anesthesia for the duration of the experiment (N = 20).

As described below, mice were sacrificed at 24 hours after TBI in order to evaluate the following parameters: 2,3,5-triphenyltetrazolium chloride (TTC) staining (N = four out of 20 for each group) [14]; histology analysis (N = three out of 20 for each group) and Tumor necrosis factor (TNF)α, Interleukin (IL)-1β, Glial fibrillary acidic protein (GFAP) and Ionized calcium binding adaptor molecule (Iba)1 immunofluorescence (N = three out of 20 for each group) [14]; Western blot analysis (N = five out of 20 for each group) and RT-PCR analysis for *Glial cell-Derived Neurotrophic Factor* (*GDNF)*, *Nerve Growth Factor* (*NGF)* and *Vascular Endothelial Growth Factor* (*VEGF)* levels (N = five out of 20 for each group). In a separate set of experiments, another 10 animals from each group were observed after TBI in order to evaluate the behavioral testing. Several recent results illustrate the importance of initiating therapeutic interventions as soon as possible following TBI, preferably within four hours post-injury, to achieve the best possible neuroprotective effect [15].

### Behavioural testing

Mice with TBI display motor and cognitive deficits. Thus, the present behavioural tests involved analyses of motor asymmetry (elevated biased swing test (EBST) and rotarod test). Training for the rotarod test was initiated at one week. Before the CCI injury, whereas no training was required for the EBST. The retard treadmill (Accuscan, Inc., Columbus, Ohio, United States) provided a motor balance and coordination assessment. Data were generated by averaging the scores (total time spent on treadmill divided by five trials) for each animal during training and testing days. Each animal was placed in a neutral position on a cylinder (3 cm and 1 cm diameter for rats and mice, respectively) then the rod was rotated with the speed accelerated linearly from 0 to 24 rpm within 60 seconds, and the time spent on the retard was recorded automatically. The maximum score given to an animal was fixed to 60. For training, animals were given five trials each day and declared having reached the criterion when they scored 60 in three consecutive trials. For testing, animals were given three trials and the average score of these three trials was used as the individual rotarod score. The EBST provided a motor asymmetry parameter and involved handling the animal by its tail and recording the direction of the biased body swings. The EBST consisted of 20 trials with the number of swings ipsilateral and contralateral to the injured hemisphere recorded and expressed in percentage to determine the biased swing activity.

### Quantification of infarct volume

Mice were anesthetized with ketamine and decapitated. Their brains were carefully removed. The brains were cut into five coronal slices of 2-mm thickness. Slices were incubated in a 2% solution of TTC at 37°C for 30 minutes and immersion fixed in 10% buffered formalin solution. TTC stains the viable brain tissue red while infracted tissue remains unstained [16,17]. For quantification of infracted area and volumes, the brain slices were photographed using a digital camera (HP Photosmart R707, Milan, Italy) and then image analysis was performed on a personal computer with an image analysis software program (using ImageJ for Windows (Institute of Mental Health, Maryland, USA). To compensate for the effect of brain edema the corrected infarct volume was calculated as previously described in detail [18]:

$$ \mathrm{Corrected}\ \mathrm{infarct}\ \mathrm{area} = \mathrm{left}\ \mathrm{hemisphere}\ \mathrm{area}\ \hbox{-}\ \left(\mathrm{right}\ \mathrm{hemisphere}\ \mathrm{area}\ \hbox{-}\ \mathrm{infarct}\ \mathrm{area}\right). $$

Values are given as mean ± SEM. The corrected total infarct volume was calculated by summing the infarct area in each slice and multiplying it by slice thickness (2 mm).

### Tissue processing and histology

Coronal sections of 5-μm thickness were sectioned from the perilesional brain area of each animal and were evaluated by an experienced histopathologist. Damaged neurons were counted and the histopathologic changes of the grey matter were scored on a six-point scale [19]: 0, no lesion observed; 1, grey matter contained one to five eosinophilic neurons; 2, grey matter contained five to 10 eosinophilic neurons; 3, grey matter contained more than 10 eosinophilic neurons; 4, small infarction (less than one third of the grey matter area); 5, moderate infarction (one third to one half of the grey matter area); 6, large infarction (more than half of the grey matter area). The scores from all the sections of each brain were averaged to give a final score for individual mice. All the histological studies were performed in a blinded fashion.

### Western blot analyses

Cytosolic and nuclear extracts were prepared as previously described [20], with slight modifications. The ipsilateral hemisphere after injury from each mouse was suspended in extraction Buffer A containing protease inhibitors, homogenized for two minutes, then centrifuged at 1,000 × g for 10 minutes at 4°C. Supernatants contained the cytosolic fraction. The pellets, containing enriched nuclei, were resuspended in Buffer B containing 1% Triton X-100, 150 mM NaCl, 10 mM Tris-HCl pH 7.4, 1 mM, ethylene glycol tetraacetic acid (EGTA), 1 mM, ethylenediaminetetraacetic acid (EDTA), 0.2 mM phenylmethanesulfonylfluoride (PMSF) and protease inhibitors. After centrifugation for 30 minutes at 15,000 × g at 4°C, the supernatants containing the nuclear protein were stored at −80°C for further analysis. The levels of inducible nitric oxide synthase (iNOS), cyclooxygenase (COX)-2, endothelial nitric oxide synthase (eNOS) and IκBα were quantified in cytosolic fractions. NFκBp65 was quantified in nuclear fractions from brain tissue collected 24 hours after TBI. The filters were probed with specific Abs anti-iNOS (1:1,000; BD Biosciences, Milan, Italy), anti-COX-2 (1:1,000; Cayman Chemicals, Tallinn Estonia), anti-eNOS (1:1000; BD Biosciences, Milan, Italy), anti-NFκBp65 (1:500; Santa Cruz Biotechnology, Heidelberg, Germany) and anti-IκBα antibody (1:500; Santa Cruz Biotechnology, Heidelberg, Germany) at 4°C overnight in 1 × PBS, 5% (w/v), non-fat dried milk and 0.1% Tween-20 (PMT). Membranes were incubated with peroxidase-conjugated bovine anti-mouse IgG secondary antibody or peroxidase-conjugated goat anti-rabbit IgG (1:2,000; Jackson ImmunoResearch, West Grove, PA, USA) for one hour at room temperature. To ascertain that blots were loaded with equal amounts of protein lysates, they were also incubated in the presence of the antibody against β-actin or lamin A/C (1:5,000; Santa Cruz Biotechnology, Heidelberg, Germany). The signals were detected with enhanced a chemiluminescence detection system reagent according to manufacturer’s instruction (Super Signal West Pico Chemiluminescent Substrate, Pierce Thermo Scientific, Rockford, IL. USA). The relative expression of the protein bands of IκBα (approximately 37 kDa), NFκB (approximately 65 kDa), eNOS (approximately 140 kDa), iNOS (approximately 130 kDa) and COX-2 (approximately 72 kDa) were quantified by densitometry with Gel Logic 200 PRO software (GE Healthcare, Milwaukee, Wisconsin, USA) and standardized to β-actin and lamin A/C levels. Images of blot signals were imported to analysis software Image Quant TL Software, version 2003 (GE Healthcare, Milwaukee, Wisconsin, USA). A preparation of commercially available molecular weight 10 to 250 kDa was used to define molecular weight positions, and as reference concentrations for each molecular weight.

### Reverse transcription polymerase chain reaction

Total RNA, from contused brain tissue at the impact site after injury, was extracted by a modified method [21], using TRIzol™ Reagent (Life Technologies, Milan, Italy) according to the manufacturer’s instructions. Reverse transcription was performed by a standard procedure using 2 μg of total RNA. After reverse transcription, 1 μl of reverse transcriptase (RT) products were diluted in 24 μl of PCR mix, to give a final concentration of 50 U ml −1 of Taq DNA polymerase (Life Technologies, Milan, Italy), 10 μm of 5′ and 3′ primers, 10 mM of each deoxynucleotide triphosphates (dNTP), 50 mM MgCl2 and 10 × NH4 buffer. cDNAs underwent 30 cycles for *GDNF*, *NGF*, *VEGF* and *β-actin*, each one performed at 94°C for one minute, melting temperature (Tm) °C for 45 seconds and 72°C for 55 seconds (Table 1). After this treatment 10 μl of RT-PCR products were separated by 1.5% agarose gel electrophoresis in Tris/Borate/EDTA (TBE) 0.5 × (Tris-base 0.089 m, boric acid 0.089 m) containing 0.1 μg ml^−1^ of ethidium bromide. Fragments of DNA were seen under ultraviolet light. *β-actin* was used as an internal reference.

**Table 1:** **Specific primer sequences**

**Gene**	**Forward primer (5′-- > 3′)**	**Reverse primer (5′-- > 3′)**
*GDNF*	TCA CTG ACT TGG GTT TGG GCT AT	TCA GAC GGC TGT TCT CAC TCC TA
*NGF*	GCA TCG AGT GAC TTT GGA GC	GTA CGC CGA TCA AAA ACG CA
*VEGF*	TGG ATG TCT ACC AGC GAA GC	ACA AGG CTC ACA GTG ATT TT
*β- actin*	CAT GAA GTG CGA CGT TGA CA	CAC ATC TGC TGG AAG GTG GA

### Immunofluorescence

After deparaffinization and rehydration, detection of TNFα, IL-1β, GFAP and Iba1 was carried out after boiling in 0.1 M citrate buffer for one minute. Non-specific adsorption was minimized by incubating the section in 2% (volume/volume (vol/vol)) normal goat serum in PBS for 20 minutes. Sections were incubated with mouse monoclonal anti-GFAP (1:100, vol/vol Santa Cruz Biotechnology (Heidelberg, Germany), or with polyclonal rabbit anti-TNFα (1:100, vol/vol Santa Cruz Biotechnology, Heidelberg, Germany), or with rabbit anti-IL-1β (1:100, vol/vol Santa Cruz Biotechnology, Heidelberg, Germany) or with mouse monoclonal anti-Iba1 (1:100, vol/vol Santa Cruz Biotechnology, Heidelberg, Germany) antibody in a humidified oxygen and nitrogen chamber for over night at 37°C. Sections were incubated with secondary antibody Fluorescein isothiocyanate (FITC)-conjugated anti-mouse Alexa Fluor-488 antibody (1:2,000 vol/vol Molecular Probes, Monza, Italy) and with TEXAS RED-conjugated anti-rabbit Alexa Fluor-594 antibody (1:1000 in PBS, vol/vol Molecular Probes, Monza, Italy) for one hour at 37°C. For nuclear staining, 2 μg/ml 4′,6′-diamidino-2-phenylindole (DAPI; Hoechst, Frankfurt, Germany) in PBS was added. Sections were observed at 20× magnification using a Leica DM2000 microscope (Leica, Milan, Italy). Optical sections of fluorescence specimens were obtained using a HeNe laser (543 nm), an ultraviolet laser (361 to 365 nm) and an argon laser (458 nm) at a one-minute, two seconds scanning speed with up to eight averages; 1.5 μm sections were obtained using a pinhole of 250. Contrast and brightness were established by examining the most brightly labeled pixels and applying settings that allowed clear visualization of structural details, while keeping the highest pixel intensities close to 200. The same settings were used for all images obtained from the other samples that had been processed in parallel. Digital images were cropped and figure montages prepared using Adobe Photoshop 7.0 (Adobe Systems; Palo Alto, California, United States).

## Materials

ATB-346 (2-(6-methoxy-napthalen-2-yl)-propionic acid 4-thiocarbamoyl-phenyl ester), sodium naproxen and TBZ (4-hydroxythiobenzamide) were provided by Antibe Therapeutics Inc. (Toronto, Canada). Unless otherwise stated, all other compounds were obtained from Sigma-Aldrich Company Ltd. (Milan, Italy). All stock solutions were prepared in non-pyrogenic saline (0.9% NaCl; Baxter, Italy) or 10% dimethyl sulfoxide (DMSO).

### Statistical evaluation

Data are mean ± SEM. Data were analyzed using Graphpad PRISM V (Graphpad Software Inc., La Jolla, California, United States). Swing activity and time on platform were analyzed using two factor repeated measures analysis of variance (RM ANOVA, group × time). Infarct area, lesion volume and densitometric analysis data were analyzed by ANOVA followed by a Bonferroni *post-hoc* test for multiple comparisons. Histological score and percentage total tissue area were analyzed by Student’s t-test. For all comparisons, *P* <0.05 was considered to be significant. In the experiments involving histology or immunohistochemistry, the figures shown are representative of at least three experiments performed on different experimental days on the tissue sections collected from all the animals in each group.

## Results

### Effect of ATB-346 on IκBα degradation and NFκBp65 translocation

**Figure 1:**
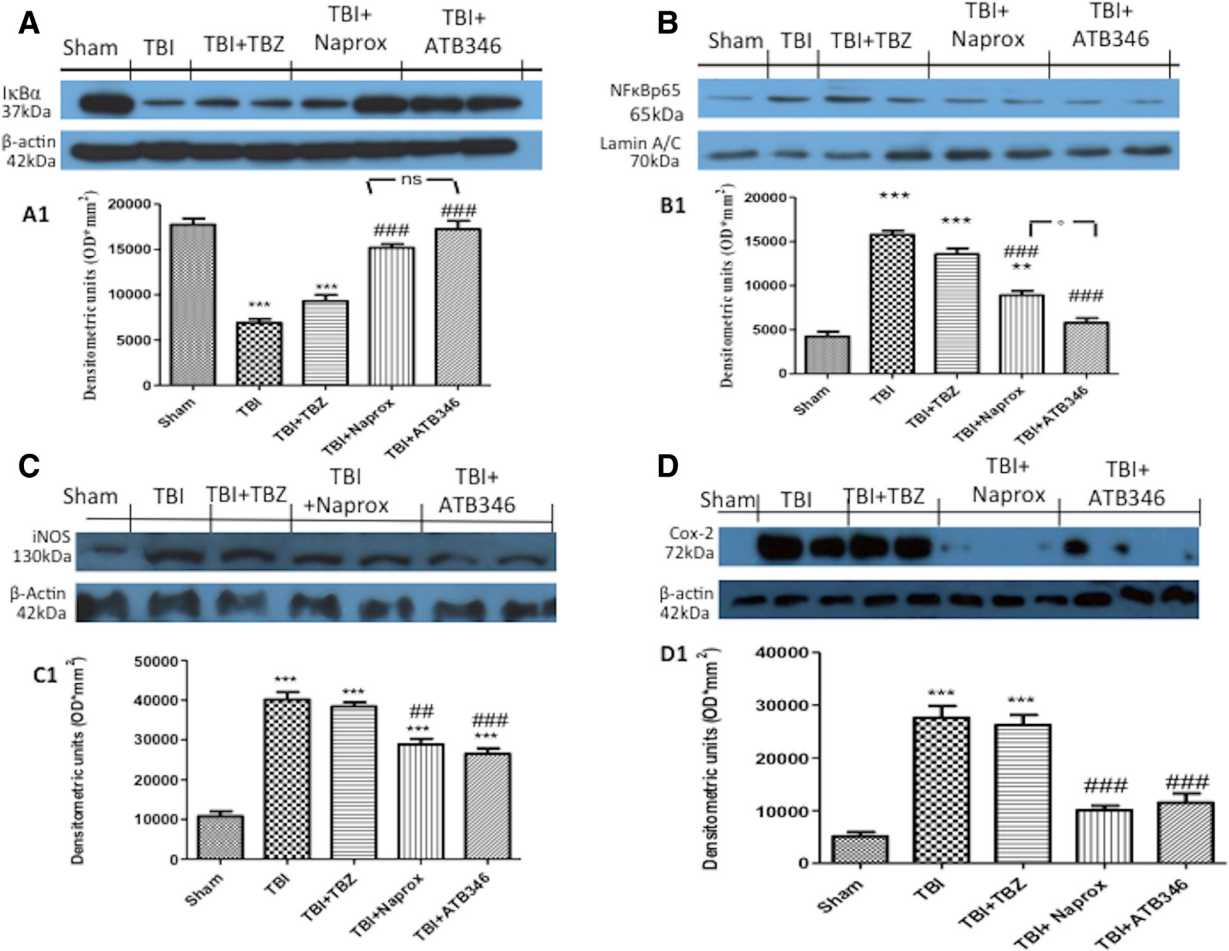
**Effects of ATB-346 on Nuclear factor κB (** **NFκB** **) pathway and pro-inflammatory enzymes.** Degradation of IκBα was significantly blocked by Naproxen and ATB-346 treatment **(A)**. Moreover, ATB-346 treatment resulted in an inhibition of nuclear translocation of p65 **(B)**. Translocation of NFκB is a critical step in the coupling of extracellular stimuli to the transcriptional activation of specific target genes. A significant increase in inducible nitric oxide synthase (iNOS) and cyclooxygenase (COX)-2 (**C** and **D**, respectively) was observed in the injured area from TBI mice compared with the Sham mice. ATB-346-treated mice had notably reduced expression of pro-inflammatory enzymes (**C** and **D**, respectively). Data show one representative blot from three independent experiments with similar results. Mean ± SEM of four to five animals per group. One-way ANOVA, followed by Bonferroni’s multiple comparison test. ***P <0.001 versus sham, ##*P* <0.01, ###*P* <0.001 versus TBI, °*P* <0.05 versus TBI + naproxen.

To investigate whether the cellular mechanism through ATB-346 could attenuate inflammatory processes we assessed Western blot analysis of the ipsilateral hemisphere after TBI, using an IκBα and an NFκB-specific antibodies. The results showed a basal expression of IκBα in the brain from sham-mice (Figure 1A, see densitometry analysis A1), while IκBα expression was significantly reduced in mice subject to TBI and TBZ administration, as showed in Figure 1A and 1A1. Naproxen treatment blunted the degradation of IκBα but ATB-346 was able to significantly restore IκBα degradation (Figure 1A, see densitometry analysis A1). Moreover, p65 subunit translocation was increased after TBI and TBZ injection in the nuclear brain homogenates, compared with sham-group. ATB-346 administration significantly reduced the translocation of p65 in nuclei compared to the TBI group (Figure 1B, see densitometry analysis B1).

### Effect of ATB-346 on iNOS and COX-2 expression

To determine the role of •NO produced during TBI, iNOS expression was evaluated by Western blot analysis. A significant increase in iNOS expression was observed in the contused area from mice subjected to TBI and TBZ administration (Figure 1C, see densitometry analysis C1). Consequently, naproxen reduced TBI-induced iNOS expression (Figure 1C, see densitometry analysis C1); on the other hand, a significant decrease in iNOS expression was observed after ATB-346 treatment (Figure 1C see densitometry analysis C1). Similarly, COX-2 expression was induced by TBI and TBZ administration compared to the sham group (Figure 1D, see densitometry analysis D1). Both treatments with naproxen and ATB-346 lowered COX-2 expression (Figure 1D, see densitometry analysis D1).

### Effect of ATB-346 on TNFα and IL-1β expression in astrocytes after traumatic brain injury

**Figure 2:**
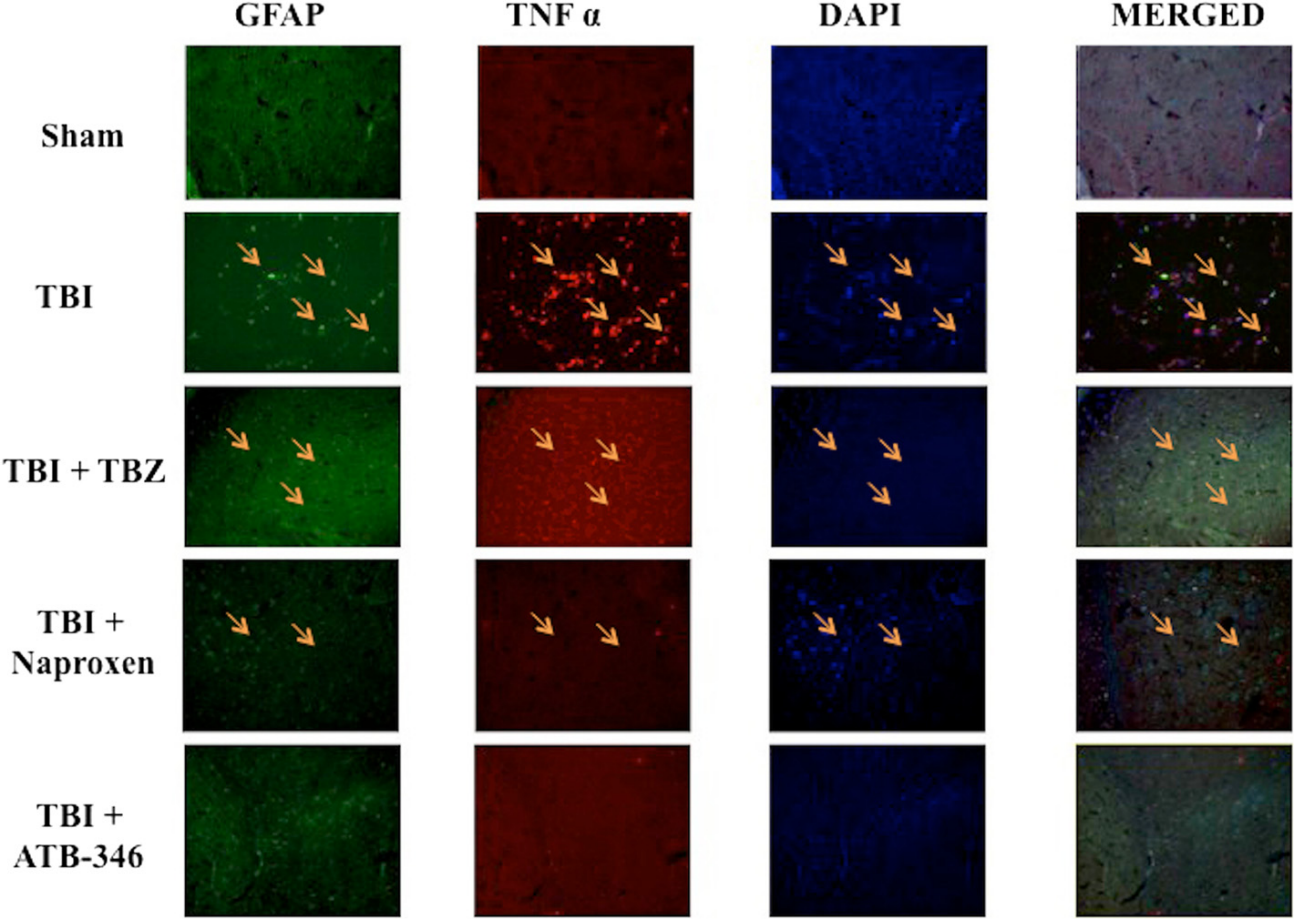
**Effects of ATB-346 on tumor necrosis factor (TNF)α expression in glial fibrillary acidic protein (GFAP) positive cells.** Brain tissue was double-stained with antibodies against GFAP, green) and TNFα (red). The red spots indicate the co-localizations (merged). Brain sections revealed increased astrogliosis (GFAP+ cells) in TBI and TBZ panels. Considerable GFAP immunoreactivity was present in TBI and TBZ panels. TNFα expression was significantly reduced by ATB-346 treatment (TBI + ATB-346 panels) compared to naproxen treatment (TBI + naproxen panels). All images were digitalized at 600 dpi.

**Figure 3:**
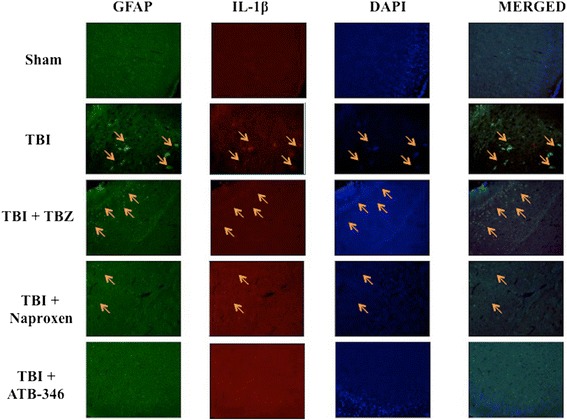
**Effects of ATB-346 on interleukin (IL)-1β expression in** **glial fibrillary acidic protein (GFAP) positive cells.** Brain tissue was double-stained with antibodies against GFAP (green) and IL-1β (red). Brain sections revealed increased astrogliosis (GFAP+ cells) in TBI and TBZ panels. Considerable GFAP immunoreactivity was present in TBI and TBZ panels. IL-1β expression was significantly reduced after ATB-346 treatment (TBI + ATB-346 panels) respect to naproxen treatment (TBI + naproxen panels). All images were digitalized at 600 dpi.

To analyse the activation of astrocytes and cytokines expression, contused brain tissue at the impact site after injury was double-stained with antibodies against GFAP (green; Figures 2 and 3) and TNFα (red; Figure 2) or IL-1β (red; Figure 3). Brain sections revealed increased astrogliosis (GFAP+ cells) in TBI and TBZ panels. Moreover, a marked co-localization of TNFα in GFAP+ cells was present after TBI and TBZ administration (merged, Figure 2). TNFα and IL-1β expressions were significantly reduced by ATB-346 treatment (TBI + ATB-346 panels; Figures 2 and 3)

.

### Effect of ATB-346 on TNFα and IL-1β expression in microglia after traumatic brain injury

**Figure 4:**
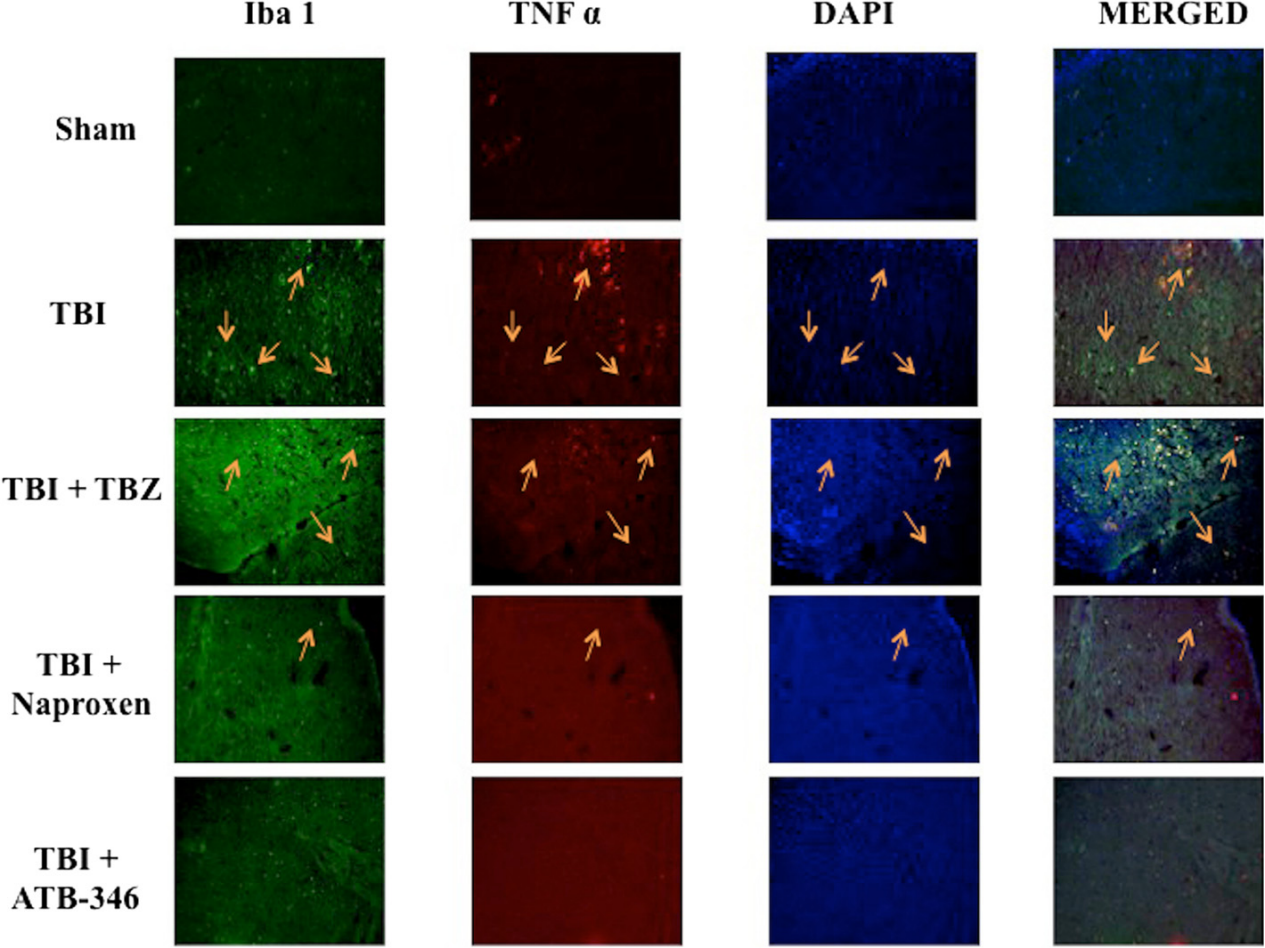
**Effects of ATB-346 on tumor necrosis factor (TNF)α in ionized calcium binding adaptor molecule (Iba)1 positive cells.** Brain tissue was double-stained with antibodies against Iba1 (green) and TNFα (red). Microglial cells (Iba1+ cells) expressed TNFα in TBI and TBZ panels. A considerable reduction of cytokine expression was present in naproxen panels; however, ATB-346 reduced notably TNFα expressions (TBI + ATB-346 panels). The red spots indicate the co-localizations (merged). All images were digitalized at 600 dpi.

**Figure 5:**
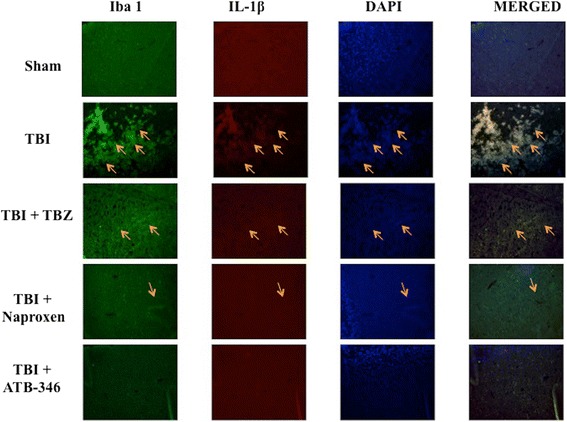
**Effects of ATB-346 on interleukin (IL)-1β expression in ionized calcium binding adaptor molecule (Iba)1 positive cells.** Brain tissue was double-stained with antibodies against Iba1 (green) and IL-1β (red). Microglial cells (Iba1+ cells) expressed IL-1β in TBI and TBZ panels. A considerable reduction of IL-1β expression was present in naproxen panels; at least, ATB-346 markedly reduced TNFα and IL-1β expressions (ATB-346 panels). The red spots indicate the co-localizations (merged). All images were digitalized at 600 dpi.

To evaluate the microglia activation and its correlation with cytokines expression, ipsilateral hemisphere to the injury site were double-stained with antibodies against Iba1 (green; Figures 4 and 5) and TNFα (red; Figure 4) or IL-1β (red; Figure 5). Microglial cells (Iba1- + cells) expressed TNFα and IL-1β in TBI and TBZ panels (Figures 4 and 5, respectively). There was an evident co-localization of TNFα and Iba1 in TBI and TBZ panels (merged, Figure 4). Considerable reductions in cytokines expressions were evident in naproxen panels (Figures 4 and 5, respectively); however, ATB-346 reduced TNFα and IL-1β expressions in microglia (Figures 4 and 5, respectively).

### Effect of ATB-346 on mRNA levels of neurotrophic factors

**Figure 6:**
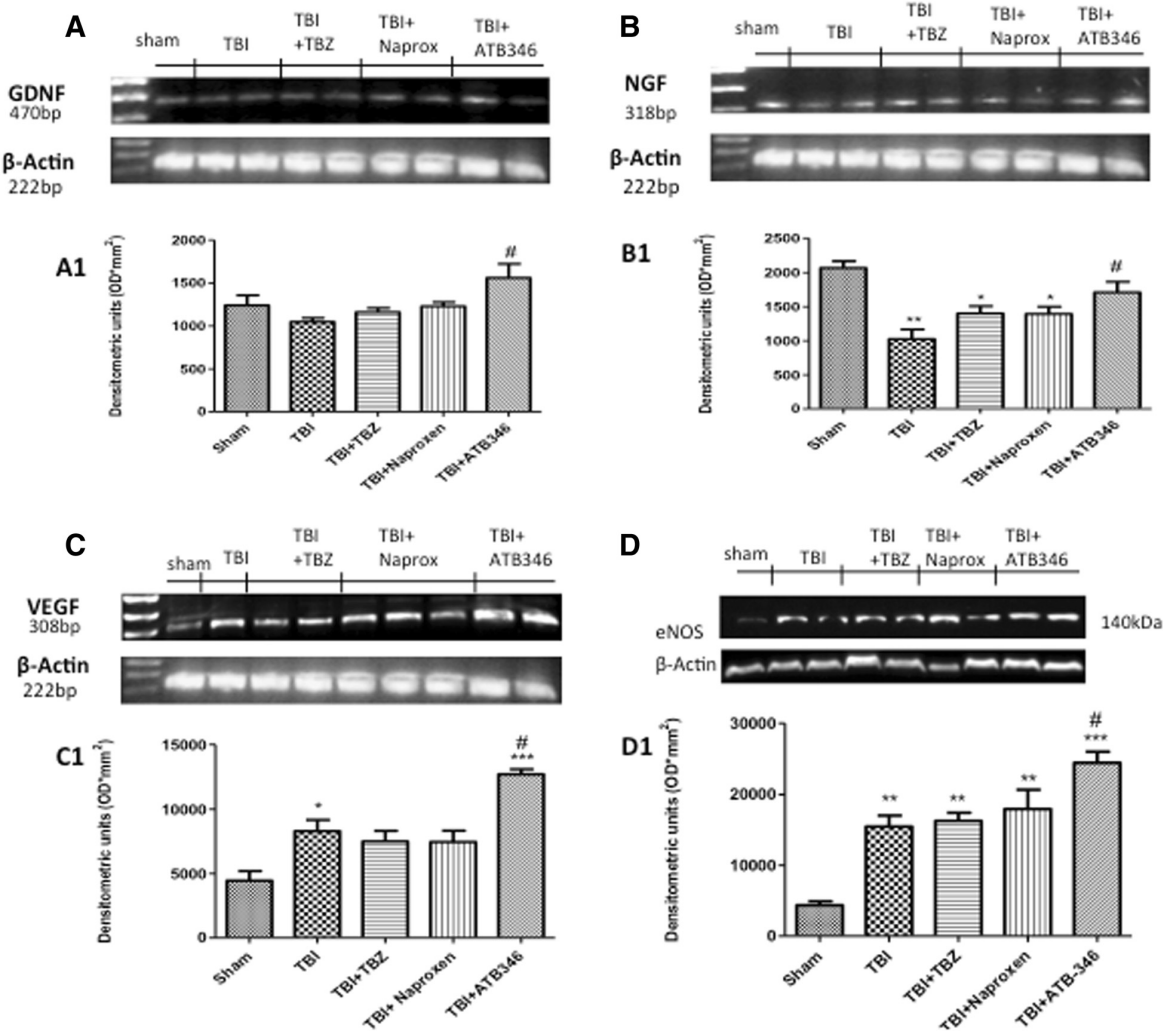
**RT–PCR analysis for** ***GDNF*** **,** ***NGF*** **and** ***VEGF*** **(A, B and C, respectively).** ATB-346 treatment significantly increased both *GDNF* and *NGF* mRNA expression compared to TBI (**A** and **B**, respectively). ATB-346 determined an important increase in *VEGF* mRNA expression **(C)**. *β-actin* was used as an internal control. mRNA was extracted and reverse-transcribed as described in the Methods section. Similar results were obtained in four additional separate experiments. No bands were observed in the absence of cDNA. Western blot analysis showed that eNOS expression in TBI mice was increased compared with sham mice **(D)**, while ATB-346 upregulated its expression **(D)**. A representative blot of homogenates obtained from five animals per group is shown, and densitometry analysis of all animals is reported (A1 to D1). A *P* value of less than 0.05 was considered significant. *P < 0.05, **P <0.01, ***P <0.001 versus sham, #*P* <0.05 versus TBI.

To test whether ATB-346 modulates the levels of the neurotrophic factors, we studied *GDNF* and *NGF* levels in brain tissue using semi-quantitative RT–PCR analysis. A significant decrease in *GDNF* (470 bp) and *NGF* (318 bp) mRNA expression following TBI and TBZ administration was evident. Moreover, ATB-346 significantly increased mRNA levels of both neurotrophic factors examined (Figure 6A and B respectively, see densitometry analysis A1 and B1 respectively).

### Effect of ATB-346 on vascular components after traumatic brain injury

To investigate whether ATB-346 could promote normalization of the impaired neurovascular unit, we observed *VEGF* level and eNOS expression. RT-PCR showed a significant increase in *VEGF* (308 bp) mRNA expression, and ATB-346 significantly increased its level (Figure 6C, see densitometry analysis C1). Moreover, by Western blot analysis we observed a significantly increase in eNOS expression in the TBI group, and ATB-346 upregulated its expression (Figure 6D, see densitometry analysis D1).

### Infarct outcome in ATB-346-treated mice after traumatic brain injury

**Figure 7:**
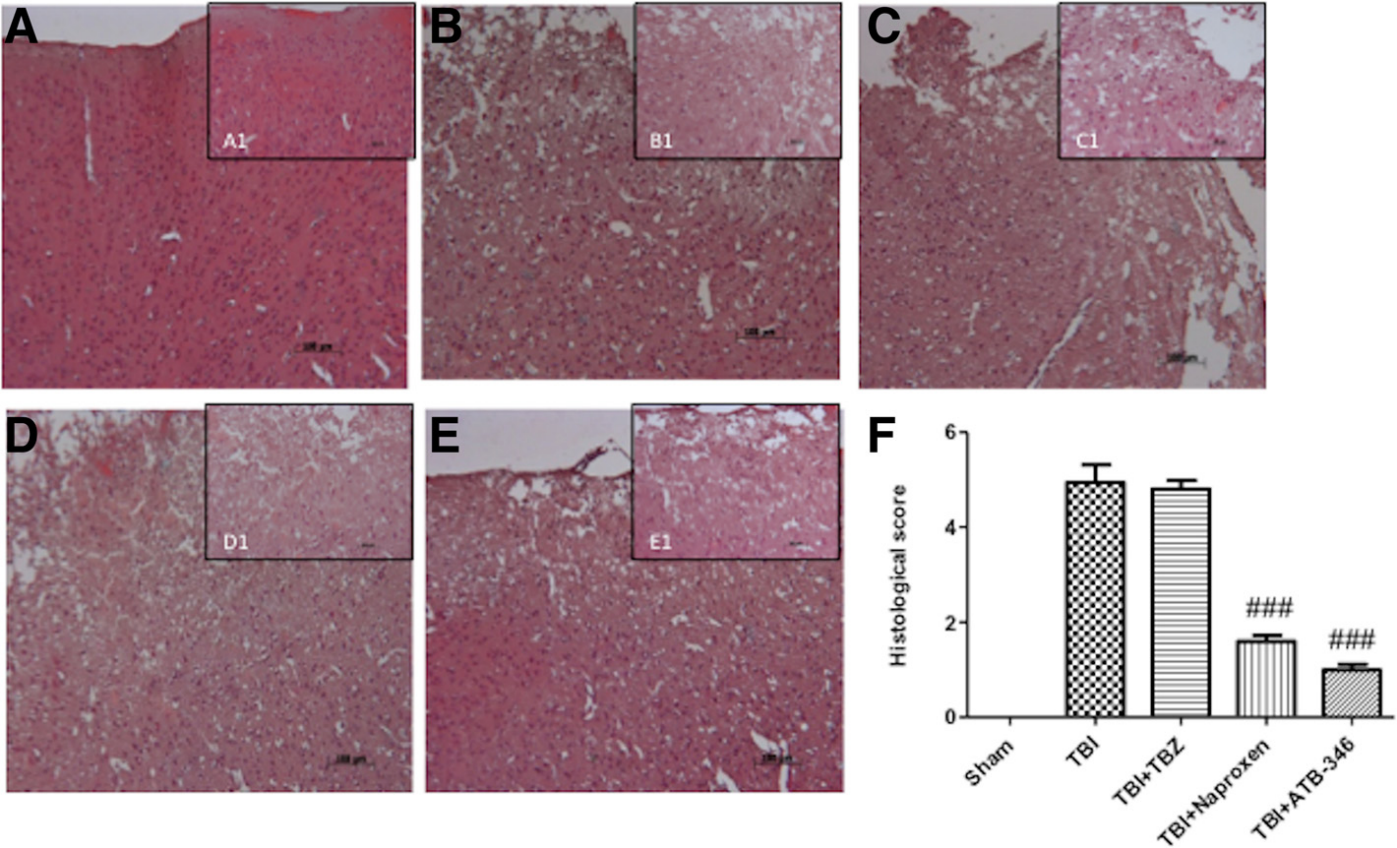
**Histological examination of brain sections after 24 hours.** Brain sections from TBI mice and TBZ-treated mice **(B** and **C** respectively, see densitometry analysis **F)** demonstrated brain tissue injury and inflammatory cell infiltration. Naproxen treatment did not attenuate completely the development of acute brain injury at one and six hours after TBI (**D**, see densitometry analysis **F**). On the contrary, ATB-346 treatment reduced the degree of brain injury and the inflammatory cells infiltration (**E**, see densitometry analysis **F**). Figure is representative of at least three experiments performed on different experimental days. ###*P* <0.001 versus TBI.

A histological examination of brain sections at the level of the perilesional area, stained 24 hours after injury, revealed significant damage in the TBI and TBI + TBZ groups, such as prominent and thickened blood vessels, ischemic changes and gliosis in the brain parenchyma (Figure 7B and C respectively, see densitometry analysis F) compared to sham mice (Figure 7A, see densitometry analysis F). Naproxen treatment attenuated the development of inflammation at 24 hours after TBI; ATB-346 significantly reduced the degree of brain injury (Figure 7D and E respectively, see densitometry analysis F).

**Figure 8:**
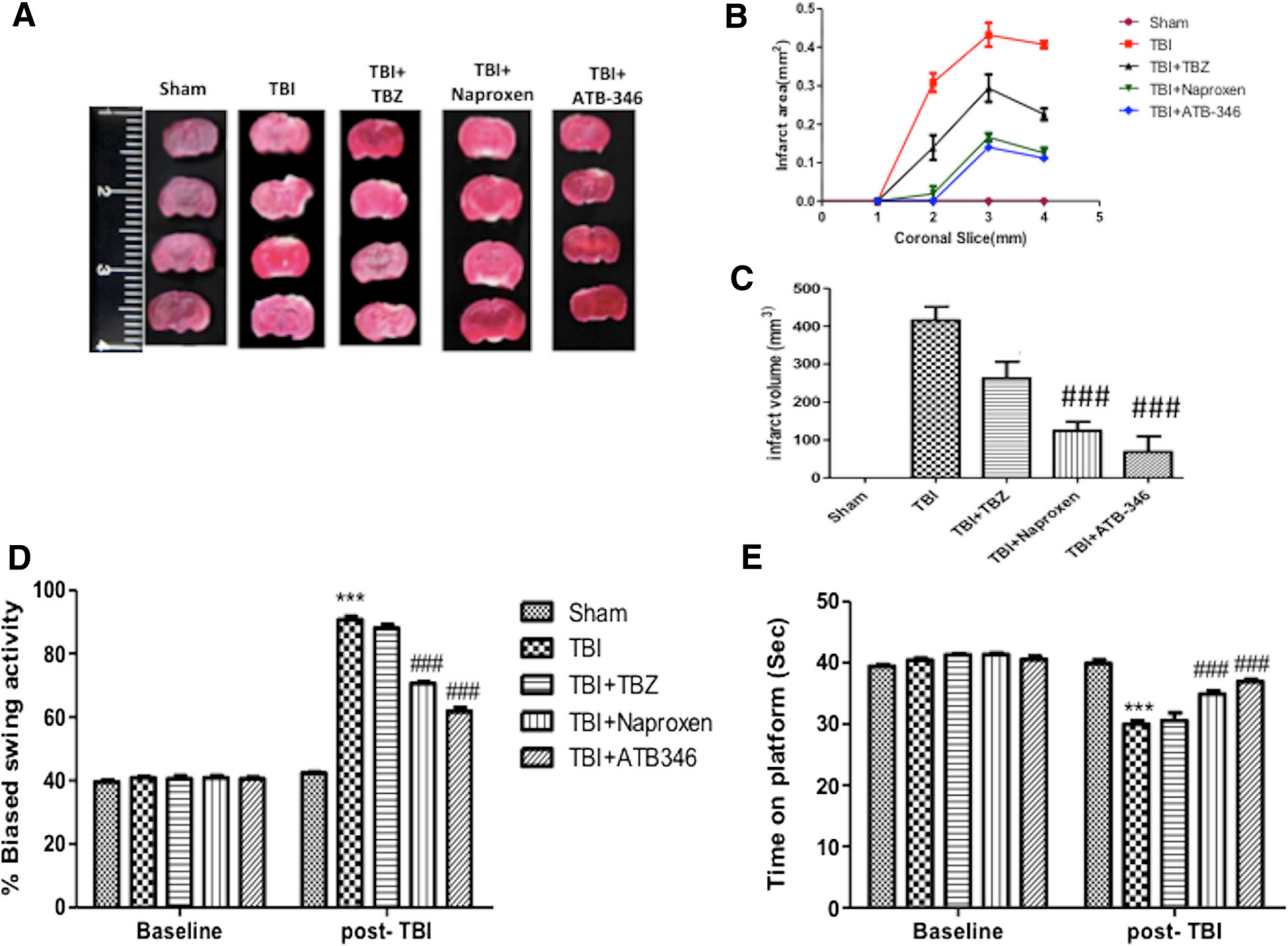
**Effect of ATB-346 on brain edema, infarction and locomotor activity.** Representative TTC stained brain section (four out of the six consecutive sections from cranial to caudate region) corresponding to largest infraction from each group **(A)**. Brain sections (2 mm thick) were stained with TTC at 24 hours after TBI and show significant difference after ATB-346 treatment in terms of area **(B)** and volume **(C)** of infarctions. The figures are representative of at least three experiments performed on different experimental days. TBI determined a range of impairments in locomotor tasks, as showed by the EBST **(D)** and the rotarod test **(E)**, after seven days. Both groups of animals that received naproxen or ATB-346 were significantly less impaired in EBST and rotarod tests compared with the TBI group. ATB-346-treated mice displayed significant improvement in their behavioral performance as revealed by decreased biased swing activity in the EBST **(D)** and increased time on the rotating rod in the rotarod test **(E)**. Each data are expressed as mean ± SEM from N = four male CD mice for each group. A *P* value of less than 0.05 was considered significant. ***P <0.001 versus sham, ###*P* <0.001 versus TBI.

Brain edema indicates pathology associated with endothelial cell activation and endothelial dysfunction. To evaluate the effect of ATB-346 on brain edema and infarctions in the TBI and TBZ group, we performed TTC staining (Figure 8A). At 24 hours after TBI, ATB-346-treated mice had a significantly smaller infarct area (Figure 8B) and volume (Figure 8C).

### Neurological deficits after ATB-346 administration

To investigate the relationship between neurological deficits in the setting of TBI we used two different tests: the EBST and the rotarod test, considered the most sensitive vestibular motor test to assess motor function. The EBST provided a motor asymmetry parameter and involved handling the animal by its tail and recording the direction of the biased body swings. The EBST consisted of 20 trials with the number of swings ipsilateral and contralateral to the ischemic hemisphere recorded and expressed in percentage to determine the biased swing activity. Mice were tested seven days after TBI for both behavioral tests. CCI-injured mice and TBZ-treated mice displayed a range of impairments in locomotor tasks as showed in Figure 8D and E, respectively. Both groups of animals that received naproxen or ATB-346 were significantly less impaired in the EBST and rotarod tests compared with the TBI group (Figure 8D and E, respectively).

## Discussion

A number of animal models have been developed to induce brain trauma. Of these the most commonly used are weight-drop injury, fluid percussion injury (FPI) and CCI. The use of TBI models has resulted in an increased understanding of the pathophysiology of TBI, including changes in molecular and cellular pathways and neurobehavioral outcomes. CCI models utilize a pneumatic pistol to laterally deform the exposed dura and provide controlled impact and quantifiable biomechanical parameters. This model produces graded, reproducible brain injury. Dependent on the severity of injury, CCI results in an ipsilateral injury with cortical contusion, hemorrhage and blood-brain barrier disruption. CCI injury reproduces changes reported in clinical head injuries such as cortical contusion, brain edema, subarachnoid hemorrhage, elevated intracerebral pressure, reduced cortical perfusion, decreased cerebral blood flow and neuro-endocrine and metabolic changes [22]. The predominantly focal brain injury caused by CCI makes this model to a useful tool for studying the pathophysiology of the secondary processes induced by focal brain injury. However, there is a lack of brain stem deformation in this model and thus a low mortality rate.

In recent years, H_2_S has been recognized as a fundamental signalling molecule that plays important roles in exerting cytoprotective effects in the CNS, since it can protect neurons and glia from oxidative stress [9,23]. H_2_S also exert many anti-inflammatory effects, including inhibition of leukocyte-endothelial adherence, reduction of edema formation [24,25] and inhibition of NFκB activation [26]. H_2_S is produced endogenously via enzymatic activity, non-enzymatic pathways (such as reduction of thiol-containing molecules), and is also released from intracellular sulfur stores (sulfane sulfur). Cystathionine β-synthase (CBS) is believed to be the critical enzyme that produces H_2_S, resulting in the modulation of neurological function. H_2_S generated by cystathionine γ-lyase (CSE) was next discovered as an important modulator of vasorelaxation in smooth muscle. They separately coordinate with L-cysteine to produce H_2_S, L-serine and ammonium. After the discovery of H_2_S as a potential neurological and vasorelaxant signaling molecule, more targets were expected to be found [27,28]. The enhanced beneficial effects of ATB-346 over those of naproxen are most likely attributable to the H_2_S release from the former, and may be due to the neuroprotective and anti-inflammatory properties of this gaseous mediator, acting in a complimentary manner to the anti-inflammatory effects associated with inhibition of COX activity. Indeed, the marked reduction of gastrointestinal toxicity of ATB-346 versus naproxen has been attributed to the mucosal protective and anti-inflammatory effects (for example, inhibition of leukocyte adherence to vascular endothelium) of the H_2_S released from this drug [29]. We observe a light beneficial effect of TBZ, the H2S-releasing moiety of ATB-346, on several parameters. The release of H_2_S from TBZ may not be as great as that from an equimolar amount of ATB-346. Previous studies have shown that TBZ alone releases very little H_2_S, but when covalently linked to another drug, such as an NSAID, considerably more H2S is released [30].

Focal lesions to the brain display a characteristic inflammatory response with infiltration of peripheral immune cells after injury. These cells are believed to be important because they contain and release a multitude of inflammatory mediators associated with increased tissue injury. Neutrophils peak approximately two days post-TBI, and monocytes slightly later [31]. Leukocyte homogenates from post-TBI patients display upregulation of iNOS, COX-2 and nicotinamide adenine dinucleotide phosphate-oxidase; all enzymes involved in producing the damaging neutrophilic oxidative burst [32]. To confirm the pathological contributions to brain inflammation, we have demonstrated here expression of COX-2 and iNOS in the injured tissue after TBI, but TBI-induced iNOS and COX-2 expression are significantly lower in injured brains from ATB-346-treated mice.

Post-TBI there is increased infiltration of neutrophils, astrocytosis, edema and both pro- and anti-inflammatory cytokines release. The major pro-inflammatory cytokines released are IL-1β, IL-6 and TNFα. The anti-inflammatory cytokines are IL-10 and transforming growth factor beta. We demonstrate that increased microglial and astrocyte activation is present 24 hours after TBI. Moreover, immunofluorescence staining showed increased TNFα and IL-1β expression in astrocytes and microglia in TBI group. ATB-346 treatment importantly reduced TNFα and IL-1β expression in these glial cells. Apparently, ATB-346 might enhance actual functional neuronal regeneration via inhibiting glial scar formation during TBI.

Neurotrophic factors have well-established roles in survival, differentiation and function of CNS neurons. Exogenous *NGF*, for example, plays a critical role in neuronal plasticity and regenerative potential, as well as the inhibition of neural apoptosis after TBI [33,34]. A study found brain-derived neurotrophic factor,(BDNF) and neurotrophin-3 (NT-3) support the survival of injured CNS neurons *in vitro* and *in vivo*, induce neurite outgrowth and increase the expression of key enzymes for neurotransmitter synthesis [35]. *GDNF* is capable of protecting against hippocampal neuronal death [36], attenuating brain swelling and reducing the lesion volume [37] after TBI. Our results visibly showed restored *GDNF* and *NGF* levels after ATB-346 treatment, maintaining their protective action.

*VEGF*, an angiogenic growth and survival factor for endothelial cells that also exhibits neurotrophic and neuroprotective effects, has been implicated in neovascularization that precedes brain tissue repair and nerve regeneration following brain injury and is required to re-establish metabolic support [38]. *VEGF* is upregulated during many pathological events, and it is induced in astrocytes located in and surrounding edematous tissue following brain contusion [39]. Our study showed that ATB-346 significantly increased *VEGF* levels. Thus, it could be hypothesized that ATB-346-facilitated the increase in *VEGF* expression in the lesion area, resulting in the secretion of *VEGF* from synthesizing cells and the restoration of the neurovascular unit.

**Figure 9:**
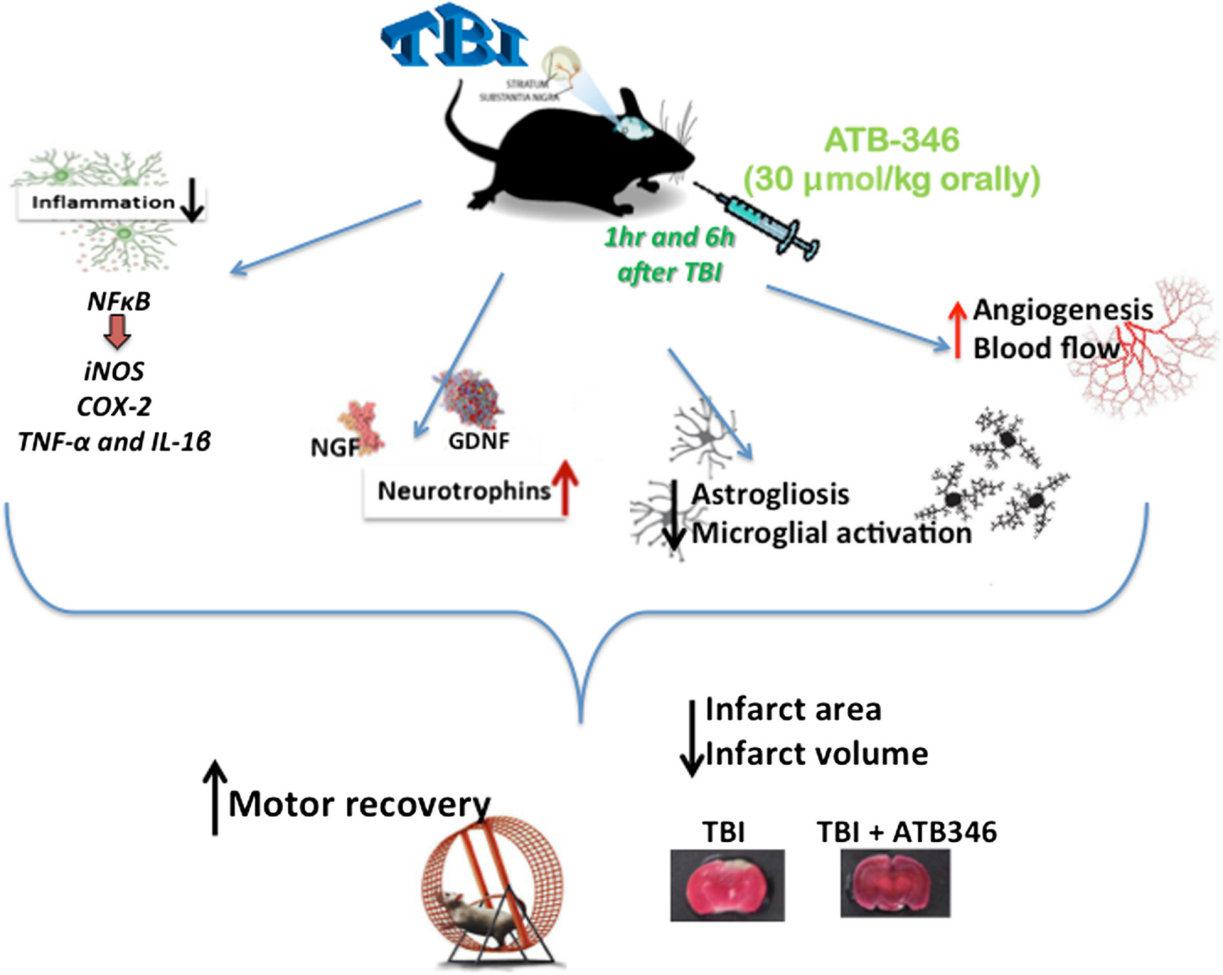
**Schematic representation of the method outlined in this experiment and results obtained.**

•NO is a key regulator of cerebral circulation by its contribution to basal vascular tone that in vasculature is mainly derived from eNOS. eNOS is predominantly expressed by vessels endothelial cells and are also located in Purkinje cell bodies in the cerebellar cortex, olfactory bulb, dentate nucleus in granular layer and hippocampal pyramidal cells and astrocytes surrounding the cerebral blood vessels [40]. A recent paper showed that eNOS is central after trauma for the maintenance of blood flow in the injured cortex for at least 24 hours after TBI; this is based on the observation that eNOS knockout mice have lower cerebral blood flow at that time point compared to wild-type mice [41]. According to this data, our results showed an evident increase in eNOS expression 24 hours after TBI. The increase in eNOS protein may represent either a protective or a reparative response, since it has been reported that eNOS is necessary to counteract posttraumatic cerebral hypoperfusion at 24 hours after CCI-TBI in mice [42]. There is substantial evidence that H_2_S upregulates •NO production in endothelial cells through the activation of eNOS, inducing angiogenesis and improving functional outcome [43-45]. Therefore, ATB 346 upregulates its expression, increasing functional protein expression and augmentation of cerebral blood flow, also in the brain. Furthermore, treatment with ATB-346 results in a significant reduction in inflammation and it is also accompanied by a detectable histological improvement of TBI. As shown in our recent paper, ATB-346 can markedly accelerate recovery of motor function in mice subjected to SCI [12]. Here, in a different model of neurotrauma, we confirmed that ATB-346 significantly improved the latency compared to the naproxen group, indicated as a mediator of the mechanism to promote recovery and to enhance the repair mechanism. In the present study, post-TBI administration of ATB-346 not only facilitated functional recovery, but also reduced tissue damage within hours following injection. The ameliorating effect of ATB-346 at tissue level was further corroborated by its ability to reduce the extent of neurodegeneration. The neuroprotective basis for these actions seems to be dependent on the H_2_S-releasing moiety of ATB-346, as also stated in a recent paper [46]. Moreover, the observation that neither naproxen nor TBZ produce the same ‘restorative’ effect on neurotrophic factors as seen with ATB-346 suggests that both suppression of COX and delivery of H^2^S is required to achieve the observed effect. The properties of this compound are summarized in Figure 9. In May 2014 Antibe Therapeutics has announced the submission of a clinical trial application to Health Canada for ATB-346.

## Conclusions

The need for developing new therapeutics for TBI treatment and the current lack of specific therapy for this indication underscore the importance of identification and characterization of novel neuroprotective compounds. Released-H_2_S may account for the absence of deleterious gastric effects, thus making of ATB-346 a potentially useful therapeutic alternative to traditional naproxen for the management of secondary damage following CNS diseases, counteracting behavioral changes and inflammatory process.

## Abbreviations

CCI: Controlled cortical impact
COX: Cyclooxygenase
EBST: Elevated Biased Swing Test
TBZ: 4-hydroxythiobenzamide
DMSO: dimethyl sulfoxide
dNTP: deoxynucleotide triphosphates
EGTA: ethylene glycol tetraacetic acid
EDTA: ethylenediaminetetraacetic acid
iNOS: inducible nitric oxide synthase
NSAIDs: Non-steroidal anti-inflammatory drugs
IL-1β: interleukin 1β
Iba1: ionized calcium binding adaptor molecule 1
TNFα: tumor necrosis factor α
GFAP: glial fibrillary acidic protein
NFκB: Nuclear factor κB
