# Correction: Hydrogen sulfide-releasing cyclooxygenase inhibitor ATB-346 enhances motor function and reduces cortical lesion volume following traumatic brain injury in mice

**DOI:** 10.1186/s12974-024-03131-6

**Published:** 2024-05-28

**Authors:** Michela Campolo, Emanuela Esposito, Akbar Ahmad, Rosanna Di Paola, Irene Paterniti, Marika Cordaro, Giuseppe Bruschetta, John L. Wallace, Salvatore Cuzzocrea

**Affiliations:** 1 Department of Biological and Environmental Sciences, University of Messina, Viale Ferdinando Stagno D’Alcontres, 31-98166 Messina, Italy; 2 Inflammation Research Network, University of Calgary, 3330 Hospital Drive NW, Calgary, AB T2N 4 N1 Canada; 3 Manchester Biomedical Research Centre, Manchester Royal Infirmary, School of Medicine, University of Manchester, 29 Grafton Street Manchester, Manchester, M13 9WU UK

**Correction: Journal of Neuroinflammation 2014, 11:196** 10.1186/s12974-014-0196-1

In this article [1], the magnification of Fig. 7 (histological evaluation) in panel B (TBI group) and C (TBI + TBZ group) were switched. Specifically, magnification B1 becomes C1 and magnification C1 becomes B1.

The revised Fig. 7 is given in this correction.

**Fig. 7:**
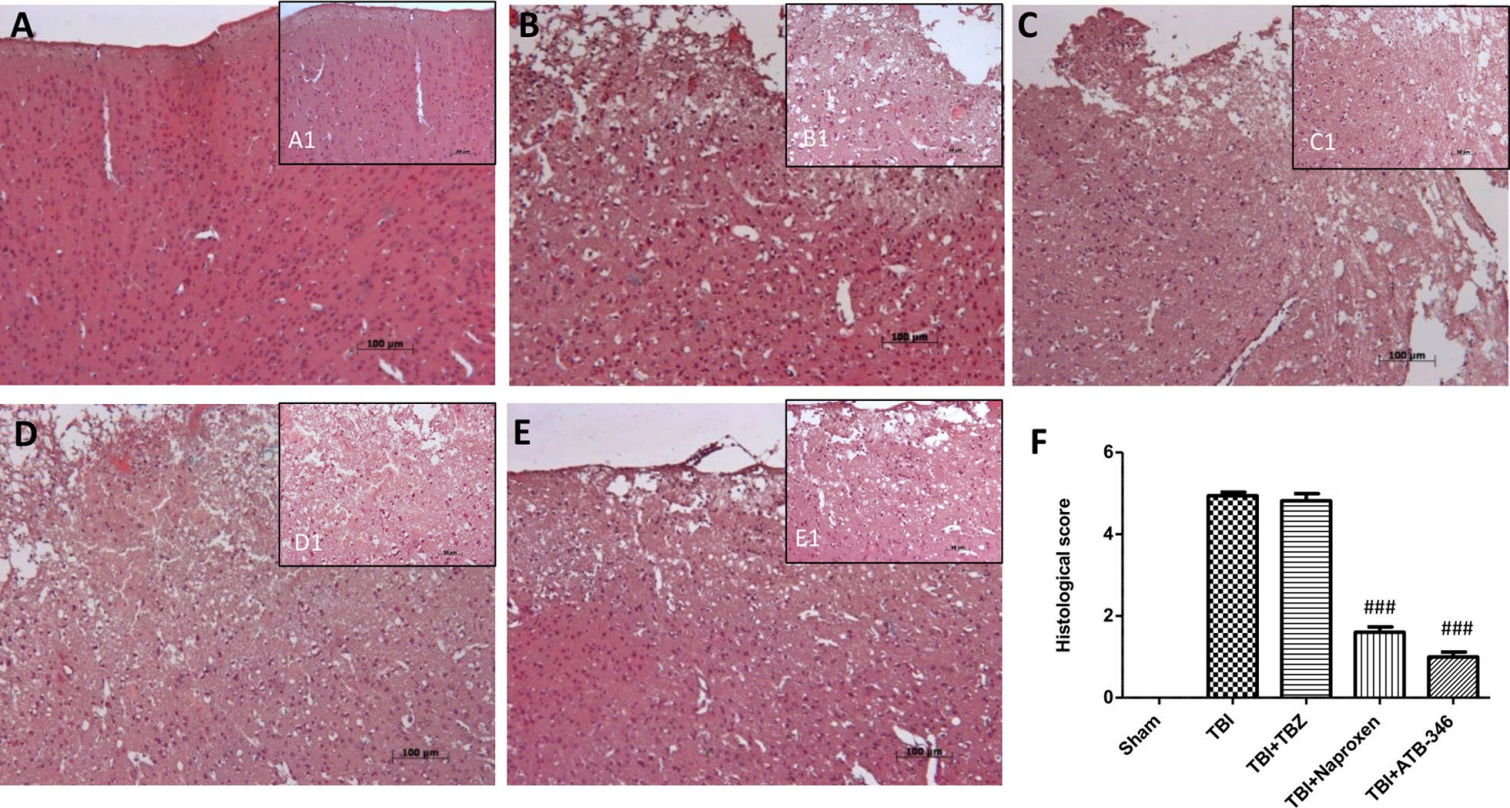
Histological examination of brain sections after 24 h. Brain sections from TBI mice and TBZ-treated mice (**B** and **C** respectively, see densitometry analysis **F**) demonstrated brain tissue injury and inflammatory cell infiltration. Naproxen treatment did not attenuate completely the development of acute brain injury at one and six hours after TBI (**D**, see densitometry analysis **F**). On the contrary, ATB-346 treatment reduced the degree of brain injury and the inflammatory cells infiltration (**E**, see densitometry analysis **F**) Sham group is represented in panel **A**. A1-E1 represent 20X magnification. Figure is representative of at least three experiments performed on different experimental days. ^###^P < 0.001 versus TBI
